# Progressive resistance training in head and neck cancer patients during concomitant chemoradiotherapy -- design of the DAHANCA 31 randomized trial

**DOI:** 10.1186/s12885-017-3388-0

**Published:** 2017-06-03

**Authors:** Camilla K. Lonkvist, Simon Lønbro, Anders Vinther, Bo Zerahn, Eva Rosenbom, Hanne Primdahl, Pernille Hojman, Julie Gehl

**Affiliations:** 1 Department of Oncology, Herlev and Gentofte Hospital, University of Copenhagen, Herlev, Denmark; 2 Department of Experimental Clinical Oncology, Aarhus University Hospital, Aarhus, Denmark; 3 Department of Public Health, Section for Sports Science, Aarhus University, Aarhus, Denmark; 4 Department of Rehabilitation, Herlev and Gentofte Hospital, University of Copenhagen, Herlev, Denmark; 5 Department of Clinical Physiology and Nuclear Medicine, Herlev and Gentofte Hospital, University of Copenhagen, Herlev, Denmark; 6 Nutritional Research Unit, Herlev and Gentofte Hospital, University of Copenhagen, Herlev, Denmark; 7 Department of Oncology, Aarhus University Hospital, Aarhus, Denmark; 8 Centre of Inflammation and Metabolism (CIM) and Centre for Physical Activity Research (CFAS), Department of Infectious Diseases, Rigshospitalet, University of Copenhagen, Copenhagen, Denmark

**Keywords:** Head and neck cancer, Head and neck squamous cell carcinoma, Chemoradiotherapy, Progressive resistance training, Exercise, Physical activity, Body composition, Lean body mass, Body weight, Weight

## Abstract

**Background:**

Head and neck cancer patients undergoing concomitant chemoradiotherapy (CCRT) frequently experience loss of muscle mass and reduced functional performance. Positive effects of exercise training are reported for many cancer types but biological mechanisms need further elucidation. This randomized study investigates whether progressive resistance training (PRT) may attenuate loss of muscle mass and functional performance. Furthermore, biochemical markers and muscle biopsies will be investigated trying to link biological mechanisms to training effects.

**Methods:**

At the Departments of Oncology at Herlev and Aarhus University Hospitals, patients with stage III/IV squamous cell carcinoma of the head and neck, scheduled for CCRT are randomized 1:1 to either a 12-week PRT program or control group, both with 1 year follow-up. Planned enrollment is 72 patients, and stratification variables are study site, sex, p16-status, and body mass index. Primary endpoint is difference in change in lean body mass (LBM) after 12 weeks of PRT, assessed by dual-energy X-ray absorptiometry (DXA). The hypothesis is that 12 weeks of PRT can attenuate the loss of LBM by at least 25%. Secondary endpoints include training adherence, changes in body composition, muscle strength, functional performance, weight, adverse events, dietary intake, self-reported physical activity, quality of life, labor market affiliation, blood biochemistry, plasma cytokine concentrations, NK-cell frequency in blood, sarcomeric protein content in muscles, as well as muscle fiber type and fiber size in muscle biopsies. Muscle biopsies are optional.

**Discussion:**

This randomized study investigates the impact of a 12-week progressive resistance training program on lean body mass and several other physiological endpoints, as well as impact on adverse events and quality of life. Furthermore, a translational approach is integrated with extensive biological sampling and exploration into cytokines and mechanisms involved. The current paper discusses decisions and methods behind exercise in head and neck cancer patients undergoing concomitant chemoradiotherapy.

**Trial registration:**

Approved by the Regional Ethics Committee for the Capital Region of Denmark (protocol id: H-15003725) and registered retrospectively at ClinicalTrials.gov (NCT02557529) September 11th 2015.

**Electronic supplementary material:**

The online version of this article (doi:10.1186/s12885-017-3388-0) contains supplementary material, which is available to authorized users.

## Background

Patients with locally advanced head and neck squamous cell carcinoma (HNSCC) undergoing concomitant chemoradiotherapy (CCRT) are often subjected to severe treatment side effects which may lead to weight loss, including loss of lean body mass, negatively impacting physical function and maybe even treatment outcome [1–7]. The loss of lean body mass (LBM) during treatment is likely to be multifactorial and HNSCC patients are particularly susceptible for several reasons: Cancer disease per se can cause muscle wasting [8, 9]; along with cisplatin chemotherapy [10, 11] and prednisolone [12, 13], which is often used as antiemetic treatment. Furthermore, many HNSCC patients fail to maintain sufficient energy and protein intake for a period of time [14, 15] due to treatment side effects, e.g. mucositis, dry mouth, pain, and fatigue. This may render patients in a catabolic state, a condition that inevitably will lead to further loss of muscle mass [16] as muscles are the largest and primary protein and energy reserve of the body. Interestingly, it has been shown that patients fail to maintain weight and LBM despite sufficient dietary intake [14], hence other interventions with potential to attenuate muscle wasting in HNSCC patients during treatment are needed.

In a preclinical study voluntary exercise efficiently mitigated cisplatin-induced muscle wasting [17]. Specifically, progressive resistance training (PRT) induces muscle hypertrophy in both healthy adults and cancer patients and definitely holds the potential to counteract cancer-related muscle wasting, too [8, 18, 19]. Twelve weeks of PRT after radiotherapy has been shown to rebuild LBM in HNSCC patients [20, 21], hence, PRT could be a meaningful approach for LBM preservation during treatment.

In a pilot study of a 12-week supervised PRT program during CCRT at our facility, we found that the intervention was feasible and appreciated by patients (Lonkvist et al., manuscript submitted). Knowing this, the present randomized trial is launched to investigate whether PRT during CCRT has a clinically relevant advantage, in terms of attenuated loss of LBM, compared with a control group not offered any structured training. In addition, extensive biological sampling is incorporated in this study adopting a translational approach, with the aim of exploring not only *if* it works, but also contributing to questions of *how* and *why*.

There is an echoing lack in clinical studies investigating the biological mechanisms. Preclinical studies demonstrate a direct inhibitory effect on cancer growth through different mechanisms [22–26]. One very plausible mechanism being exercise-mediated induction of intratumoral natural killer cells (NK cells) [27], unequivocally linking exercise to attenuation of tumor growth in mice [28, 29].

Exercise in its broadest sense is a very heterogeneous activity making the description of exercise interventions in clinical trials critical [30]. This article describes the study design of a 12-week progressive resistance training program in head and neck cancer patients undergoing concomitant chemoradiotherapy, sharing thoughts behind the decision making.

## Methods/design

### Design

**Fig. 1:**
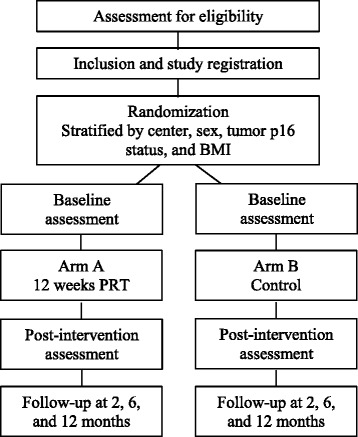
Overall study design

In this prospective phase II multi-center randomized study in patients with HNSCC scheduled for radiotherapy concomitant with chemotherapy (cisplatin), the effects of 12-week PRT are investigated. The study is planned to include 72 patients from the departments of oncology at Aarhus and Herlev Hospitals in Denmark, see study flow in Fig. 1. Also, a third center was opted to participate but this site will not be including patients due to capacity issues.

Ethics approval has been obtained from the regional Ethics Committee for the Capital Region of Denmark (H-15003725) and the Danish Data Protection Agency (HGH-2015-003; 2005–41-4802; 2014–41-3510). The study is registered at clinicaltrials.gov (NCT02557529) September 11th 2015. This article describes protocol version 4.0 from April 1st 2016.

The manuscript applies to the SPIRIT guidelines of randomized trials. The SPIRIT checklist, appendix for the SPIRIT checklist, as well as the World Health Organization (WHO) Trial Registration Data Set can be found in additional files 1, 2 and 3.

### Participants

Patients are eligible if the following inclusion criteria are fulfilled: 1) Histologically verified primary head and neck squamous cell carcinoma (HNSCC) of the oral cavity, oropharynx, hypopharynx, larynx, or in lymph nodes of the neck from an unknown primary tumor; 2) candidate for curatively intended CCRT (weekly cisplatin during radiotherapy, 66–68 Gy) according to Danish Head and Neck Cancer (DAHANCA) group (i.e. patients with stage III-IV disease, www.dahanca.dk) [31]; 3) performance status (PS) 0–1 (Eastern Cooperative Oncology Group Performance (ECOG); 4) age ≥ 18 years; 5) signed informed consent.

Exclusion criteria are: 1) Body Mass Index (BMI) < 20.5; 2) comorbidity potentially interfering with attendance or test results, e.g. other cancers, diabetes, prednisolone treatment); 3) tonsillectomy within 1 week before inclusion; 4) psychological, social or geographical conditions that could influence protocol adherence; 5) insufficient bone marrow function (hemoglobin <6 mmol/L, leucocytes <2.5 × 10^9^/L, or thrombocytes <50 × 10^9^/L; 6) diastolic blood pressure < 45 or >95, resting heart rate > 100; 7) signs of ischemia on electrocardiogram; 8) pregnancy.

### Randomization

Patients will be stratified by site (Herlev/Aarhus), sex (male/female), p16-status of the tumor (positive/negative) and BMI (<30/≥30) and randomized 1:1 to either a training group performing a 12-week PRT program or control group. If a patient leaves the study within the first week, the number of patients randomized will be increased by one. Patient inclusion form is faxed to The Danish Head and Neck Cancer (DAHANCA, Aarhus, Denmark) group administration, that performs the randomization using a software randomization file (developed and used by the DAHANCA group) that automatically and randomly allocates each patient in either group. The personnel conducting the randomization are independent of clinical personnel and are not otherwise involved in the study.

### Treatment

All patients will receive curatively intended CCRT, 66 to 68 Gy, in 2 Gy fractions, 6 fractions/week, with concurrent nimorazole perorally (1200 mg/m^2^) [32] before each fraction (1000 mg/m^2^ for same day second fraction), and weekly cisplatin (40 mg/m^2^, max. 70 mg). Prophylactic antiemetics are administered according to institutional guidelines (Additional file 4: Table S1).

### Intervention

The PRT program comprises seven conventional resistance training exercises targeting the large muscle groups of the body (chest press, low row (Herlev site)/lateral pull down (Aarhus site), hamstring curls, knee extension, leg press, abdominal crunches (Herlev site)/sit ups (Aarhus site), back extensions) (Additional file 5: Figure S1). The latter two included primarily to ensure a full body workout. The training protocol (Table 1) was tested in our pilot study (Lonkvist et al., manuscript submitted) and is almost identical to the protocol developed and used in the DAHANCA 25 trials [20, 21]. The first week is an introductory week with high repetition number and low load, as many HNSCC patients are resistance training naïve. The intensity and volume progressed throughout the program from two to three sets with a load corresponding to 15 to 8 repetition maximum (RM), i.e. the load that can be lifted respectively 15 to 8 times using proper technique (See Tables 1 and 2). This progression model is in accordance with the guidelines from the American College of Sports Medicine (ACSM) [33]. Due to the focus on muscular hypertrophy, patients are urged to perform all sets in all exercises to exhaustion within the given RM target, thereby ensuring local muscular fatigue. In accordance, patients are instructed to perform all sets of each exercise before moving on to the next exercise and also to perform the lower body exercises after each other before moving to upper body exercises. Each exercise is executed in full range of motion and with rest periods of no more than 60 s between sets (Table 2). If more repetitions than planned can be performed, the training load will be increased to match the specific RM target. Training sessions are planned three times a week every other day ensuring optimal recovery time for maximal hypertrophic response. In case of temporary discontinuation, patients will proceed with the same weight as when they paused, but will be adjusted to ensure the proper RM target is reached.

**Table 1:** Exercise progression model

Training session	Repetitions	Sets
1–3	15	2
4–6	12	2
7–18	12	3
19–31	10	3
32–36	8	3

**Table 2:** Description of the PRT program

Load	15 RM (week 1), 12 RM (week 2–6), 10 RM (week 7–10), 8 RM (week 11–12)
Repetitions	15 (week 1), 12 (week 2–6), 10 (week 7–10), 8 (week 11–12)
Sets per sessions	2 (week 1–2), 3 (week 3–12)
Sessions per week	3
Duration of training period	12 weeks
Rest between sets	45–60 s
Rest between repetitions	0 s
Range of motion	Maximum possible
Rest between training sessions	Training every other day

Abbreviations: *PRT* progressive resistance training, *RM* repetition maximum, e.g. 15 RM is the heaviest load that can be lifted 15 times using proper technique. Sec, seconds

The PRT program starts concurrently with CCRT. 36 training sessions are planned, i.e. thrice weekly for 12 weeks, thus continuing approximately 6 weeks further than CCRT. If a session is cancelled due to treatment related interventions, radiotherapy, scans, or due to public holidays, the session will be replaced at the end of the training program. Sessions missed for personal reasons or incapacitation will not be substituted. It will be ensured that the PRT program and tests never compromise treatment schedule.

At the Herlev site conventional exercise equipment is used for all exercises but due to different equipment available at the involved sites, three specific exercises differ but with only minimal difference in target muscle groups or progression options. Thus, at the Aarhus site sit-ups will be performed as traditional floor exercises with free weights ensuring possible progression in intensity. Hamstring curls will be performed using elastic bands (TheraBand, The Hygenic Corporation, Ohio, USA) with varying resistance and lateral pull down replaces low seated row in Aarhus. Conventional resistance training machines (Technogym, Gambettola, Italy) are used at both sites.

During the first 6 weeks all training sessions are supervised by physiotherapist or educated training instructors. When possible the same supervised training modality at the hospital training facility will continue for the remaining 6 weeks. If a patient is unable to attend training sessions at the hospital, e.g. due to prolonged transport time, the remaining training sessions will be tailored individually at commercial training facilities near the patients’ own home. However, patients must attend at least one session per week supervised at the hospital training facility.

If patients, due to treatment side effects, are unable to attend at least one weekly training session, they will be given a leaflet describing two simple exercises (backward lunges and push-ups), and they will be encouraged to do the exercises (3 sets, 12 repetitions) every day until they are able to attend the supervised training again. Patients will fill in training logs during every session from which training adherence, changes in training volume, and intensity are reported.

No direct criteria for discontinuing the training program are provided, but if a patient feels incapable of training or if the physician, physiotherapist or training instructor deems the patient’s general condition not compatible with training, the program will be paused.

To support energy intake and mitigate negative energy balance on training days, patients are offered a meal and/or a protein supplement (e.g. Nutridrink compact (Nutricia), 125 mL, 1260 kJ, 12 g protein) immediately after training sessions. Patients in both groups are continuously screened (i.e. body weight assessments) by trained nurses ensuring the best possible energy intake to limit the risk of catabolic state during the CCRT and the approximately 6-week follow-up period immediately after.

### Controls

No restrictions on physical activity (PA) or other concomitant care are made for the control patients but no organized training will be offered to them. PA is reported in training logs. Except for blood sampling which, obviously, will not be drawn after any training session (see below) in the control group, there are no differences in tests and assessments between the groups.

### Study objectives and assessments

The primary endpoint is change in lean body mass (LBM). Secondary endpoints are training adherence and changes in and difference between groups in body composition, muscle strength, functional performance, weight, adverse events, dietary intake, self-reported PA, quality of life (QoL), labor market affiliation, blood biochemistry, cytokines in plasma, NK-cells in peripheral blood, sarcomeric protein content in muscles, as well as muscle fiber type and fiber size in muscle biopsies. See Table 3 for all assessment time points.

**Table 3:**
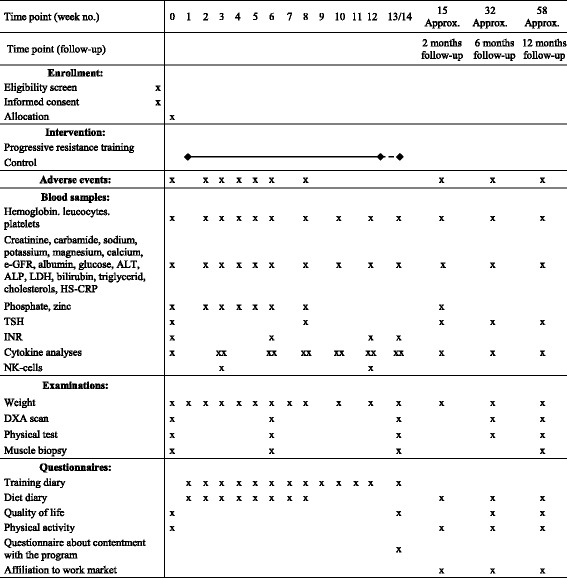
Time schedule for study assessments

X marks when an examination is planned. XX marks at which time points blood samples are drawn both before and after a training session in the training group. Regarding week 13/14: Due to public holidays training can extend beyond 12 weeks, training continues until 36 sessions have been offered. Blood samples are drawn, weight registered and other examinations are performed after the 36 sessions. Abbreviations: ALT, alanine aminotransferase. ALP, alkaline phosphatase. e-GFR, estimated glomerular filtration rate. LDH, lactate dehydrogenase. HS-CRP, high-sensitive c-reactive protein. TSH, thyroid stimulating hormone. INR, international normalized ratio. DXA, dual-energy X-ray absorptiometry. Approx., approximately

### Clinical outcomes

#### Body composition

The primary endpoint is change in lean body mass (LBM) which will be assessed after 12 weeks of PRT or control using Dual Energy X-ray Absorptiometry (DXA) (Herlev site: GE lunar iDXA, GE Healthcare Technologies; Aarhus site: Hologic QDR-series, Hologic Inc., Bedford, MA, USA). The hypothesis is that 12 weeks of progressive resistance training can attenuate the loss of LBM by at least 25%. This time span was chosen since 12 weeks are considered a sufficient period of PRT needed to affect LBM. Furthermore, this time point is a usual evaluating point for head and neck cancer as it coincide with the 2-month post-radiotherapy follow-up. Changes in total body mass and fat mass will also be assessed. Total body weight, with patients in light clothing and no shoes, will be measured weekly by the same digital scale at each site during therapy and bi-weekly thereafter.

#### Adherence

Adherence to the PRT program is registered by the physiotherapist or educated training instructors. Adherence to the study in general is encouraged by highlighting the importance of both groups in order for the trial to produce valid results. Furthermore, most appointments are planned when patients already have an appointment at the hospital, making it as convenient as possible for the patients.

#### Maximal muscle strength

All physical tests are conducted by the physiotherapist or the training instructors. Muscle strength will be evaluated by 1RM test of unilateral leg press (dominant leg) and bi-lateral chest press performed in the conventional equipment used in training. One RM tests are widely used when evaluating changes in maximal muscle strength in cancer patients [34, 35]. Following an exercise specific warm up, the patient will have one attempt with a given load, which will gradually be increased until the patient is unable to lift the load throughout a standardized range of motion using proper technique. As few attempts as possible will be used and a two-minute rest is ensured between all attempts to limit the risk of muscular fatigue.

#### Functional performance

Functional performance resembling activities of daily living will be evaluated using the 30 s chair stand test, 30 s arm curl test, and maximal stair climbing performance, best of two attempts. These are frequently used in cancer patients, including patients with HNSCC [20].

#### Treatment side-effects

Adverse events will be monitored according to Common Terminology Criteria for Adverse Events (CTCAE) version 4.0 [36], performance status will be registered according to ECOG scale, and pain using the Numeric Rating Scale (NRS) for pain, consisting of 11 points from 0 (no pain) to 10 (worst pain imaginable) [37].

#### Cytokine analyses, standard blood samples, and NK-cells

Standard blood samples will be taken according to schedule (Table 3). Blood samples for cytokine analyses will be taken before and after training sessions according to schedule (Table 3). Based on comprehensive explorative analyses with samples from a primary cohort of patients (Lonkvist et al., manuscript submitted), we have identified a list of particularly interesting cytokines and other molecules for further analyses, including 6Ckine/chemokine (C-C motif) ligand 21 (CCL21), cutaneous T cell-attracting chemokine (CTACK)/CCL27, interleukin 6 (IL-6), IL-8/CXCL8, IL-15, IL-16, monocyte chemoattractant protein 1 (MCP-1)/CCL2, MCP-2/CCL8, macrophage-derived chemokine (MDC)/CCL22, macrophage migration inhibitory factor (MIF), macrophage inflammatory protein-1α (MIP-1α)//CCL3, thymus-expressed chemokine (TECK)/CCL25, tumor necrosis factor α (TNF-α), soluble epidermal growth factor receptor (sEGFR), basic fibroblast growth factor (FGF-basic), follistatin, hepatocyte growth factor (HGF), leptin, platelet-derived growth factor AB/BB (PDGF-AB/BB), prolactin, stem cell factor (SCF), soluble vascular endothelial growth factor-1 (sVEGFR-1), and sVEGFR-2. In initial analyses some of these cytokines increased during CCRT whilst others decreased, and the interesting point would be to investigate if PRT may affect these changes in either direction. Furthermore, the mobilization of NK-cells during PRT will be evaluated in week 3 and 12. The frequency and cytotoxic profile of the NK-cells will be analyzed by flow cytometry by staining for the surface receptors CD3, CD16 and CD56, as well as intracellular expression of Granzyme B and Ki-67.

#### Muscle biopsies

Muscle biopsies are optional for patients, but if accepted, they will be collected under sterile conditions from the middle lateral part of the vastus lateralis muscle using a 5 mm Bergstrom biopsy cannula preceded by local anesthesia (lidocaine 10 mg/ml). Both satisfactory thrombocyte count (≥ 40 × 10^9^/L) and International Standard Ratio (≤ 1.5) will be confirmed. Biopsies are taken from the mid-thigh of the same leg but a few centimeters from the previous biopsy at each time point to avoid variation between legs in the analyses. The samples will be dissected to be free of visible fat and connective tissue. A well-aligned portion of the biopsy for muscle fiber morphology analyses will immediately mounted in Tissue-Tek (Qiagen, Valencia, CA) and frozen in isopentane precooled in liquid nitrogen. The rest of the biopsy for later proteomics analyses will be frozen directly in liquid nitrogen. All samples will be stored at −80 °C until analysis.

The muscle biopsies are planned to be used for investigating differences in changes in muscle fiber types, protein expression, and metabolic pathways between the two groups.

### Questionnaires

#### Physical activity

To register PA in addition to the supervised training of the PRT group as well as all PA in the control group, patients will fill in a weekly trial specific questionnaire on PA. Thus, type of activity (running, resistance training, walking etc.) and the daily duration of the activity will be registered every week from baseline to the end of the training period. Also, patients will fill in a physical activity scale (PAS) questionnaire for measuring average weekly PA of sleep, work, and leisure time [38].

#### Energy intake

Patients will receive dietary counseling by clinical dietician before or immediately after start of treatment as well as by educated nurses during the treatment period. If patients are admitted due to nutritional issues during the treatment period, they will be seen by a clinical dietician. Resting metabolic rate (RMR) will be estimated using the Mifflin-St. Jeor formula described elsewhere [39]. Energy expenditure (kilojoules per day) is measured as: Energy need (kJ) = RMR x activity factor × 4.184. Activity factor will be based on self-reported PA at the different time points. Protein need will be estimated as 18% of total energy need: Protein need (gram) = (total energy need (kJ) × 18)/17. A clinical dietician will calculate total daily energy intake based on patient reported information from a questionnaire filled in weekly during treatment (Table 3). The number of patients needing tube feeding and the duration of the tube feeding will also be registered.

#### Quality of Life (QoL)

Changes in QoL will be evaluated using the European Organisation for Research and Treatment of Cancer (EORTC) quality of life questionnaires, QLQ-C30 and QLQ-H&N-35, which have previously been used in exercise studies in cancer survivors [40] and in Danish HNSCC patients [41].

#### Satisfaction with the program

A semi-structured questionnaire was developed asking patients to grade the effect the program have had on their physical, psychological, and social well-being on a scale from 1 to 10, 1 being “very positively”; 10 being “very negatively”. Furthermore, patients are asked if scheduling was convenient and whether the PRT program was appropriate, too hard or too light (training group only). In addition, they can make free text on all questions.

#### Work

Affiliation to work market will be registered as a measure of convalescence, measuring how soon patients return to work and to what extent. At 2, 6, and 12 months follow-up patients fill in a questionnaire with information about current work status, date when work was resumed, and at 2 months follow-up, also, educational status, occupation, and work hours prior to diagnosis will be registered.

### Blinding procedures

Assessment of the primary endpoint (LBM) will be blinded since the personnel performing DXA scans will not be aware of randomization status of the patients. Due to practicalities physical tests cannot be blinded. However, tests are standardized and performed by the same personnel regardless of randomization. Personnel analyzing blood samples and muscle biopsies are blinded to patient identity and group allocation.

### Statistical considerations

The primary endpoint is difference in mean change of LBM between the training and the control group, specifically, whether attending this particular PRT program can significantly attenuate the loss of LBM after 12 weeks of PRT, which approximately aligns with the time where the treatment is evaluated (2 months after end of radiotherapy). Thus, the time at which the primary endpoint is evaluated is at 12 weeks after initiation of PRT.

The second important decision was to define a clinically meaningful endpoint in terms of difference in change in LBM loss. Of course, the 12 week period in which it was possible to train was included as a parameter since the short training period would influence the possible outcome, and even more so as patients were expected to lose LBM as a result of side effects to treatment. Limited clinical data were available on the possible effect of a 12-week PRT program on LBM change during concomitant chemoradiotherapy, though Lonbro et al. did find that head and neck cancer patients attending a 12-week PRT program initiated after radiotherapy gained an average of 2.3 kg (95% CI 1.7–3.0) [20]. A similar effect can probably not be expected when PRT is performed during chemoradiotherapy as the patients during this time are in a catabolic state. This was seen in our pilot study where patients despite PRT had a mean LBM loss of 3.6 kg (Lonkvist et al., manuscript submitted).

Based on these deliberations we concluded that a difference of 25% in LBM loss between the exercise group and the control group (which, estimated from data in the pilot study, would be 1.2 kg of LBM in absolute difference) would be a clinically meaningful difference, and yet, an obtainable goal in the circumstance of undergoing CCRT.

Thus, the sample size calculation is based on changes in LBM in our one-armed pilot study where a 3.6 kg reduction (corresponding to 6.8%) in LBM was detected after 12 weeks of PRT (Lonkvist et al., manuscript submitted). A priori, a sample size of 34 in each group will have 80% power to detect a difference in means of 25% ((9.06%–6.8%)/9.06%) between the two groups (corresponding to an estimated mean LBM loss of 6.80% from baseline in the training group and an estimated LBM loss of 9.06% in the control group with a standard deviation of 3.27). A two group t-test with a 0.05 two-sided significance level was used.

An anticipated drop-out rate of 5% is included in the calculations to ameliorate the risk of inadequate patient number for analyses, hence 36 patients are planned to be enrolled in each group (total *n* = 72). Patients dropping out before or during the first week of treatment will be replaced by another patient.

Analyses will include descriptive analyses as well as mixed model repeated measures analyses examining differences between groups and over time. The α-level of statistical significance will be set to 0.05.

## Discussion

An increasing body of evidence underlines the numerous benefits of physical exercise in terms of improving patient wellbeing and rehabilitation after cancer therapy, and very interestingly a tumor-inhibiting effect of exercise is being unraveled [27, 42–47].

In particular head and neck cancer patients experience perturbing loss of lean body mass and are severely affected by treatment for weeks and months after completion [1, 48]. Thus, there is ample reason to investigate a possible beneficial role of exercise during head and neck cancer treatment but at the same time exercise studies require careful attention to a number of issues. In particular, when investigating exercise in patients undergoing CCRT a number of specific challenges must be addressed. We designed a randomized trial on exercise for head and neck cancer patients undergoing concomitant CCRT and decided to describe the strategy in this article about the protocol.

The choice of primary endpoint, as well as the assessment hereof, should be cautiously chosen. We chose difference in change in LBM at the 12-week assessment, as it has been shown that weight loss, especially loss of LBM, may negatively affect physical function, morbidity and mortality in patients undergoing CCRT [5, 7]. Thus, it would be interesting to investigate if PRT may ameliorate LBM loss, and 12 weeks are often considered a minimum amount of time for an effect on LBM by PRT. Furthermore, a standard evaluation time point in head and neck cancer patients is at this time as it almost coincides with the 2-month post CCRT assessment. At this time evaluation of the effect of treatment is performed and side-effects have often diminished substantially.

The chosen assessment method is conventional DXA scan based on several factors: Compared to other methods it is a low risk, precise measurement of whole body composition [49–52] where data may be retrieved and reevaluated at a later time point, if needed. Furthermore, it is fast and relatively inexpensive.

In this study we are only including patients receiving an intense treatment with concomitant chemoradiotherapy. The patients have the most intense treatment schedule, hence, if it possible for them to attend and benefit from the program, there is no reason to assume that the findings may not be relevant for head and neck cancer patients receiving other radiotherapy regimens.

Prescribing exercise in a training intervention study could be thought of as prescribing medicine, where it is indisputable that specifications such as type, dose, interval, and duration of treatment are essential information in reporting. In this trial, PRT is the obvious choice of training modality, with the primary endpoint being change in LBM. Exercise intensity, volume and frequency are chosen to ensure optimal progression throughout the program and are based on previous studies in HNSCC patients [20, 21] as well as guidelines from the American College of Sports Medicine (ACSM) [33]. Training days and number of sessions per week are chosen to ensure adequate rest between sessions for optimal hypertrophy response in the muscles. Likewise, patients are instructed to exercise to exhaustion for maximal hypertrophic response. In general, all training sessions are supervised by physiotherapists or educated training instructors. However, for practical reasons, an exception is allowed, i.e. it will be possible for the last 6 weeks to train at a public center closer to home, and only attend supervised training once a week to ensure progression. Unsupervised training holds the advantage of flexibility for both patient and caregiver team, while on the other hand, supervised sessions are a necessity to ensure that prescribed dose is executed and reported correctly.

To ensure faster enrollment the study is conducted at two institutions which can compromise standardization of the PRT program. Three specific exercises differ, but cautions are taken so that it should not influence primary outcome notably.

An essential aspect to consider is the fact that these patients have a very busy schedule in regards to treatment. Radiotherapy six times a week, combined with chemotherapy treatment once a week, as well as appointments with doctors and nurses make planning a challenge. Treatment delays are deleterious to outcome [53], thus, planning training sessions, tests, and scans conveniently so they do not interfere with treatment is crucial. Furthermore, seeing to that patients do not have too strenuous days and that meals are offered are also significant factors for attendance and thus effect of the intervention. The patients often suffer from side effects, e.g. fatigue, nausea, and xerostomia, which are likely to be limiting factors in any intention to exercise. Hence, optimizing schedule is crucial for several reasons and should be carefully managed.

Sufficient protein and energy supply is vital for muscle growth or preservation. Hence, reporting on the effect of any training modality in HNSCC patients must include reporting of dietary intake, too. In this study customized diet diaries are completed regularly by patients. These diaries also form a basis for starting a conversation about diet and advised strategy for the patient, e.g. about tube feeding when necessary.

Anemia during radiotherapy is associated with response to treatment [54–56] which is an important factor to consider when planning blood sampling. Patients receive weekly cisplatin, hence blood sampling is done weekly, evaluating hematology. If hemoglobin is low blood sampling for research purposes will be paused. Furthermore, blood sampling for cytokine analyses are planned so that only a maximum of 42 mL of extra blood is drawn during the entire treatment period, while more frequent sampling is done in the approximately 6 weeks after treatment.

Blood samples will be used for explorative analyses of the differences between the groups in regards to cytokines and cancer markers over time. Furthermore, the PRT group will have samples taken before and after training sessions to investigate whether a bout of resistance training will release myokines and NK-cells as it is known from endurance exercise [27, 57, 58]. Doing this is important to contribute to the investigation of the biological mechanisms behind a possible effect. Muscle biopsies are optional in order for it not to be a limiting factor for patient recruitment. When performing samples on a part of patients, bias can be a concern, however, in our pilot study 2/3 of patients accepted muscle biopsies (Lonkvist et al., manuscript submitted).

It is planned to investigate the effect of resistance training on changes in muscle fiber types, protein expression, and metabolic pathways between the two groups.

Several patient-reported outcome measures are also evaluated in this study, including QoL and PA. Exercise has been shown to increase QoL in cancer patients [59, 60], and exercise studies in HNSCC patients confirm the positive effect [61–64]. Still, it is relevant to include QoL measures in new intervention studies, if the type of intervention or program differs from prior programs. EORTC QLQ-30 and H&N-35 questionnaires have been chosen to evaluate QoL, these questionnaires are validated and often used in cancer research [2, 59]. PA is evaluated using the validated PAS questionnaire [38] to assess level of PA in both groups since an apparent bias is that patients in the control group might start to exercise regularly, thereby affecting the between group differences.

To evaluate patients’ satisfaction with the program, we developed a semi-structured questionnaire asking patients about the effect of the program on their physical, psychological, and social well-being. We have added this to set a direct reaction on what patients felt about the program.

An inherent bias in all exercise trials is that patients who have less comorbidity and are in better performance status may be more inclined to accept participation. In our pilot study all patients had p16-positive (HPV-associated) tumors (Lonkvist et al., manuscript submitted). Patients with p16-positive tumors have been shown to have larger disease stages, averagely less tobacco and alcohol consumption, fewer comorbidities, and be in better performance status [65]. Further studies will be needed to look at the effects of exercise programs in patients with poor performance status, comorbidities, or with a history of tobacco or alcohol consumption.

Designing and performing exercise trials in cancer patients requires careful consideration to optimal modality, dose, duration, and many other parameters depending on desired outcome measures. Also, and at least as important, is a detailed description in order to ensure clarity and reproducibility. Finally, interpretation of biological sampling for mechanistic investigations must be recommended and these samples, in connection with the clinical data, may help to generate important knowledge. With this study, we hope to contribute with influential results regarding progressive resistance training in head and neck cancer patients undergoing concomitant chemoradiotherapy.

## Additional files

Additional file 1: SPIRIT checklist. The SPIRIT checklist. (PDF 44 kb)

Additional file 2: Appendix A for SPIRIT checklist. Appendix A for SPIRIT checklist. (PDF 170 kb)

Additional file 3: Appendix B for SPIRIT checklist. WHO Trial Registration Data Set. (PDF 895 kb)

Additional file 4: Table S1. Antiemetic regimens. Antiemetics are given according to institutional guidelines. At Herlev site the regimen changed May 23rd 2016 due to standardization in the Capital Region. Day 1 is the day cisplatin is given. In addition, Domperidon 20–30 mg PRN is administered P.O. a maximum of thrice daily. Abbreviations: p.o., per os. PRN, pro re nata (when necessary). (PDF 14 kb)

Additional file 5: Figure S1. Illustrations of the exercises. Illustrations of the exercises. (PDF 24 kb)

## Abbreviations

ALP: Alkaline Phosphatase
ALT: Alanine Aminotransferase
BMI: Body Mass Index
CCRT: Concomitant Chemoradiotherapy
DAHANCA: Danish Head and Neck Cancer
DXA: Dual-energy X-ray Absorptiometry
e-GFR: Estimated Glomerular Filtration Rate
EORTC: European Organisation for Research and Treatment of Cancer
FM: Fat Mass
HNSCC: Head and Neck Squamous Cell Carcinoma
HPV: Human Papillomavirus
HS-CRP: High-Sensitive C-Reactive Protein
INR: International Normalized Ratio
KJ: Kilojoules
LBM: Lean Body Mass
LDH: Lactate Dehydrogenase
P.O.: Perorally
PA: Physical Activity
PAS: Physical Activity Scale
PRN: Pro Re Nata (when necessary)
PROM: Patient Reported Outcome Measure
PRT: Progressive Resistance Training
QLQ: Quality of Life Questionnaire
QoL: Quality of Life
RM: Repetition Maximum
RMR: Resting Metabolic Rate
TBM: Total Body Mass
TSH: Thyroid Stimulating Hormone
