# Supplementary material for: Progressive resistance training in head and neck cancer patients during concomitant chemoradiotherapy -- design of the DAHANCA 31 randomized trial
Source: BMC Cancer. 2017 Jun 3;17:400. doi: 10.1186/s12885-017-3388-0 (PMC5457597; doi:10.1186/s12885-017-3388-0)
Supplement: Supplementary file 1 — SPIRIT checklist. The SPIRIT checklist. (PDF 44 kb) [file 12885_2017_3388_MOESM1_ESM.pdf]

Appendix A. SPIRIT 2013 Checklist – item no.:

2a. See printed version from www.clinicaltrials.gov.

5b. Julie Gehl M.D., Dr. Med. Sci.

Department of Oncology

Herlev and Gentofte Hospital, University of Copenhagen, Herlev Ringvej 75, 2730 Herlev, Denmark

E-mail: Karen.Julie.Gehl@regionh.dk

Phone: +45 38683868, paiger no 82981

15. All referrals are checked for eligibility. If eligible, the patient is informed about the protocol by a doctor at the first appointment. Furthermore, the patient receives written information and is given time to read and consider whether he/she wants to participate.

16c. Eligible patients are informed by a physician at their first appointment and are given time to consider participation. If they accept, the patient inclusion form is sent by fax to The Danish Head and Neck Cancer (DAHANCA, Aarhus, Denmark) group administration, who performs the randomization and faxes the allocation group back to the treating physician who will inform the patient about group allocation.

18a. Patient care (treatment, training and testing) is performed by a small group of physicians, nurses, physiotherapists or training instructors, all trained for their specific task relating to the protocol.

Data collection forms are in Danish and can be obtained from the trial sponsor by request.

18b. The protocol has one year follow-up after completed therapy. Afterwards patients continue standard follow-up for another four years. Hence, follow-up is already planned which eases the protocol's follow-up. In Denmark everybody has a social security number, hence only very rarely do patients "disappear". They do follow the planned follow-up. Furthermore, the importance of follow-up is emphasized to the patient. No data will be collected for patients who choose to leave the protocol.

19. Data management has been approved by the Danish Data Protection Agency and will be collected and stored accordingly. DXA data entry will be double-checked since this is the primary endpoint.

20c. Protocol non-adherence will be registered (i.e. Number of training sessions of 36 offered sessions). All patients in the training arm will be analyzed as being in the training arm, regardless of attended sessions. Mixed model repeated measure analyses are chosen to handle missing data.

21a. DMC is not needed since it is a non-medical intervention, hence, data monitoring is not mandatory and was not opted.

21b. No interim analyses planned. No stopping guidelines have been made because it is a non-medical intervention that is considered safe.

22. Patients follow standard procedures during the entire protocol which means that patients are seen weekly by a physician during the treatment period and CTC registration is done. After the treatment period patients are seen after one week (by a nurse), after two weeks (by a physician), and after 2 months (by a physician). Patients in the training arm are seen at least once weekly by a physiotherapist of training specialist. Furthermore, all patients are told to call in if anything unusual happens.

23. No auditing is planned due to non-medical intervention.

25. Important protocol changes (amendments) will not be implemented without approval from the regional Ethics Committee for the Capital Region of Denmark. The centers are in close contact and any changes will be communicated directly between relevant parties.

26a. See 16c.

26b. The Ethics Committee and the Danish Data Protection Agency have approved biological samples to be saved for any ancillary studies. These may not be conducted, though, without new approval from the Ethics Committee.

27. Any data and personal information is collected, shared, and maintained according to approval from the Danish Data Protection Agency.

29. Trial investigators have access to the final trial dataset. If relevant, it will be available by request to investigators.

30. In Denmark, there is free health care, hence, no provisions are offered. In case of any harm suffered due to intervention or tests, patient’s insurance can be applied for.

31a. Trial results will be published in English language, peer-reviewed journal. Patients will be offered summary of trial results in Danish, as well as any published literature in English. Furthermore, all patients are allowed access to their own trial results.

31b. Professional writers will not be used. Investigators that meet the International Committee of Medical Journal Editors (ICMJE) recommendations will co-author.

32. Consent form, as well as all other information to the patients about the protocol, is in Danish and is available if requested.

33. Biological specimens are collected and stored according to approval from the regional Ethics Committee for the Capital Region of Denmark and the Danish Data Protection Agency. No future studies may be conducted without new approval from the Ethics Committee.
