# Supplementary material for: Progressive resistance training in head and neck cancer patients during concomitant chemoradiotherapy -- design of the DAHANCA 31 randomized trial
Source: BMC Cancer. 2017 Jun 3;17:400. doi: 10.1186/s12885-017-3388-0 (PMC5457597; doi:10.1186/s12885-017-3388-0)
Supplement: Supplementary file 2 — Appendix A for SPIRIT checklist. Appendix A for SPIRIT checklist. (PDF 170 kb) [file 12885_2017_3388_MOESM2_ESM.pdf]

ClinicalTrials.gov
A service of the U.S. National Institutes of Health

Progressive Resistance Training in Head and Neck Cancer Patients During Concomitant Chemoradiotherapy

This study is currently recruiting participants. (see Contacts and Locations)

Verified May 2016 by Herlev Hospital

Sponsor:
Herlev Hospital

Collaborators:
Aarhus University Hospital
Odense University Hospital
Danish Head and Neck Cancer Group

Information provided by (Responsible Party):
Julie Gehl, Herlev Hospital

ClinicalTrials.gov Identifier:
NCT02557529

First received: September 11, 2015
Last updated: May 16, 2016
Last verified: May 2016
History of Changes

Tracking Information

**First Received Date** ICMJE	September 11, 2015
**Last Updated Date**	May 16, 2016
**Start Date** ICMJE	August 2015
**Estimated Primary Completion Date**	December 2017 (final data collection date for primary outcome measure)
**Current Primary Outcome Measures** ICMJE (submitted: September 22, 2015)	Change in LBM (lean body mass) [Time Frame: at 12-weeks post PRT] [Designated as safety issue: No]<br>change in kilograms
**Original Primary Outcome Measures** ICMJE	*Same as current*
**Change History**	Complete list of historical versions of study NCT02557529 on ClinicalTrials.gov Archive Site
**Current Secondary Outcome Measures** ICMJE (submitted: May 16, 2016)	• Change in LBM (lean body mass) [Time Frame: at 6 weeks, and 6 and 12 months post RT] [Designated as safety issue: No]<br>change in kilograms<br>• Fat mass [Time Frame: at 6 and 12 weeks, and 6 and 12 months post RT] [Designated as safety issue: No]<br>change in kilograms<br>• Weight loss [Time Frame: at 6 and 12 weeks, and 6 and 12 months post RT] [Designated as safety issue: No]<br>in kilograms<br>• pain [Time Frame: at 6 and 12 weeks, and 6 and 12 months post RT] [Designated as safety issue: Yes]<br>measured by NRS-scale<br>• Quality of Life [Time Frame: at 6 and 12 weeks, and 6 and 12 months post RT] [Designated as safety issue: No]<br>QLQ-C30 questionnaire<br>• Muscle strength [Time Frame: at 6 and 12 weeks, and 6 and 12 months post RT] [Designated as safety issue: No]<br>measured using chest press and leg press

	• Chair rise [Time Frame: at 6 and 12 weeks, and 6 and 12 months post RT] [Designated as safety issue: No]<br>measured using 30 s. chair rise<br>• arm curls [Time Frame: at 6 and 12 weeks, and 6 and 12 months post RT] [Designated as safety issue: No]<br>measured using 30 s. arm curls<br>• Stair climb [Time Frame: at 6 and 12 weeks, 6 and 12 months] [Designated as safety issue: No]<br>steps/sec<br>• Compliance to PRT program [Time Frame: at 12 weeks post PRT] [Designated as safety issue: No]<br>No. of attended sessions out of total<br>• Physical activity [Time Frame: at 6 and 12 weeks, and 6 and 12 months post RT] [Designated as safety issue: No]<br>measured by PAS (physical activity scale)<br>• Percent of patients with feeding tubes [Time Frame: at 6 and 12 weeks, and 6 and 12 months post RT] [Designated as safety issue: Yes]<br>• Resumption of work [Time Frame: At 12 months follow-up] [Designated as safety issue: No]<br>No. of days from end of radiotherapy until back at work at full time (or the hours of work per week as before therapy)<br>• Percent relapses [Time Frame: at 12 months follow-up] [Designated as safety issue: Yes]<br>Percent of patients having relapse within 12 months after radiotherapy<br>• Cytokines [Time Frame: at 3, 6, 8, 10, and 12 weeks] [Designated as safety issue: No]<br>Measurement of different cytokines, changes over time and after bout of PRT. Will be measured regularly during the 12 weeks of PRT.<br>• Muscle biopsies [Time Frame: at 6 and 12 weeks, and at 12 months follow-up] [Designated as safety issue: No]<br>protein will be measured using the proteomic approach. Muscle fiber type and size will be evaluated.<br>• Patient satisfaction [Time Frame: at 12 weeks] [Designated as safety issue: No]<br>Study specific questionnaire regarding pros and cons of attending the study<br>• NK-cells [Time Frame: at 3 and 12 weeks] [Designated as safety issue: No]<br>Measuring NK-cells. NK-cells may increase after exercise and show tumor inhibiting effect.
**Original Secondary Outcome Measures** ICMJE<br>**(submitted: September 22, 2015)**	• Change in LBM (lean body mass) [Time Frame: at 6 weeks, and 6 and 12 months post RT] [Designated as safety issue: No]<br>change in kilograms<br>• Fat mass [Time Frame: at 6 and 12 weeks, and 6 and 12 months post RT] [Designated as safety issue: No]<br>change in kilograms<br>• Weight loss [Time Frame: at 6 and 12 weeks, and 6 and 12 months post RT] [Designated as safety issue: No]<br>in kilograms<br>• pain [Time Frame: at 6 and 12 weeks, and 6 and 12 months post RT] [Designated as safety issue: Yes]<br>measured by NRS-scale<br>• Quality of Life [Time Frame: at 6 and 12 weeks, and 6 and 12 months post RT] [Designated as safety issue: No]<br>QLQ-C30 questionnaire<br>• Muscle strength [Time Frame: at 6 and 12 weeks, and 6 and 12 months post RT] [Designated as safety issue: No]<br>measured using chest press and leg press<br>• Chair rise [Time Frame: at 6 and 12 weeks, and 6 and 12 months post RT] [Designated as safety issue: No]<br>measured using 30 s. chair rise<br>• arm curls [Time Frame: at 6 and 12 weeks, and 6 and 12 months post RT] [Designated as safety issue: No]<br>measured using 30 s. arm curls<br>• Stair climb [Time Frame: at 6 and 12 weeks, 6 and 12 months] [Designated as safety issue: No]<br>steps/sec

	• Compliance to PRT program [Time Frame: at 12 weeks post PRT] [Designated as safety issue: No]<br>No. of attended sessions out of total<br>• Physical activity [Time Frame: at 6 and 12 weeks, and 6 and 12 months post RT] [Designated as safety issue: No]<br>measured by PAS (physical activity scale)<br>• Percent of patients with feeding tubes [Time Frame: at 6 and 12 weeks, and 6 and 12 months post RT] [Designated as safety issue: Yes]<br>• Resumption of work [Time Frame: At 12 months follow-up] [Designated as safety issue: No]<br>No. of days from end of radiotherapy until back at work at full time (or the hours of work per week as before therapy)<br>• Percent relapses [Time Frame: at 12 months follow-up] [Designated as safety issue: Yes]<br>Percent of patients having relapse within 12 months after radiotherapy
**Current Other Outcome Measures** ICMJE	*Not Provided*
**Original Other Outcome Measures** ICMJE	*Not Provided*

Descriptive Information

**Brief Title** ICMJE	Progressive Resistance Training in Head and Neck Cancer Patients During Concomitant Chemoradiotherapy
**Official Title** ICMJE	Progressive Resistance Training in Head and Neck Cancer Patients During Concomitant Chemoradiotherapy - The **DAHANCA** 31 Study
**Brief Summary**	72 patients with head and neck cancer, undergoing primary treatment with radiation therapy and concomitant weekly cisplatin, will be recruited to this multicentre trial.<br>Randomized 1:1 to either 12-week progressive resistance training (PRT) program or control arm, starting together with concomitant chemoradiotherapy (CCRT) Stratified by centre, gender, p16-status and body mass index (BMI) below or above 30.<br>Primary endpoint is difference in change in lean body mass (LBM) between the groups and the endpoint is reduction of LBM loss in intervention arm by 25% compared to control.<br>Secondary endpoints include side-effects to treatment, change in body composition, physical function and strength, and compliance to PRT. Questionnaires on QoL, diet, voluntary exercise and work affiliation will also be registered.<br>Blood samples for explorative analyses will be drawn and optional muscle biopsies drawn for proteomics analyses and histological analyses.
**Detailed Description**	The PRT program will start about the onset of radiotherapy. The program consists of 7 exercises in training machines and involves the major muscle groups of the body.<br>The program has previously been found to successfully restore the loss of lean body mass (LBM) in head and neck cancer patients post-treatment. A group based approach will be used to facilitate a social and motivating training environment for the patients. A pilot study (NCT02068950) showed feasibility of PRT during CCRT.<br>In addition to baseline data (height, tumor stage, performance status, etc), the following parameters will be registered: Weight, patient reported side effects, as well as a questionnaire on amount of physical activity and food intake.<br>Physical function and strength will be tested at baseline, after the course of chemoradiotherapy and at the end of the 12-week PRT programme, and at 6 and 12 months follow-up. DXA scans for body composition will be performed using a Lunar iDXA (GE Healthcare).<br>Blood samples will be drawn at regular intervals during the 12 weeks and at follow-up.<br>Patient reported quality of life (EORTC Quality of Life Questionnaire (QLQ)-C30 and QLQ-H&N35) will also be registered.<br>Muscle biopsies will be taken three times: at baseline, after treatment and after 12-weeks PRT.<br>Study duration is expected to be 18 months and an additional 12 months for follow-up.
**Study Type** ICMJE	Interventional
**Study Phase**	Phase 2
**Study Design** ICMJE	Allocation: Randomized<br>Endpoint Classification: Efficacy Study

	Intervention Model: Factorial Assignment<br>Masking: Open Label<br>Primary Purpose: Supportive Care
**Condition** ICMJE	• Head and Neck Neoplasms<br>• Weight Loss
**Intervention** ICMJE	• Behavioral: Progressive Resistance Training<br>12 weeks supervised resistance training program. Details of the program: 12 weeks, 3 sessions per week, 7 exercises in training machines (leg press, leg curl, hamstring curl, chest press, lateral pull down, sit-ups and back extensions). In general 2-3 sets of 8-15 repetitions will be performed following a progression plan starting with more repetitions at lower intensity progressing to fewer repetitions at higher intensity during the 12-week period (American College of Sports Medicine Position Stand)<br>• Behavioral: physical activity<br>Weekly diary of performed physical activity using the Physical Activity Score (PAS) during the 12-weeks intervention<br>Other Name: Diary of performed physical activity<br>• Behavioral: Diet diary<br>Weekly diet diary during the 12-weeks intervention
**Study Arm (s)**	• Experimental: Progressive Resistance Training<br>12 weeks progressive resistance training (PRT) during and after concomitant chemoradiotherapy. Also optional/voluntary physical activity performed on their own is registered, as well as diet diary.<br>Interventions:<br>◦ Behavioral: Progressive Resistance Training<br>◦ Behavioral: physical activity<br>◦ Behavioral: Diet diary<br>• Active Comparator: Control<br>Control arm. Optional/voluntary physical activity performed on their own is registered, as well as diet diary.<br>Interventions:<br>◦ Behavioral: physical activity<br>◦ Behavioral: Diet diary
**Publications** *	*Not Provided*

*** Includes publications given by the data provider as well as publications identified by ClinicalTrials.gov Identifier (NCT Number) in Medline.**

Recruitment Information

**Recruitment Status** ICMJE	Recruiting
**Estimated Enrollment** ICMJE	72
**Estimated Completion Date**	May 2018
**Estimated Primary Completion Date**	December 2017 (final data collection date for primary outcome measure)
**Eligibility Criteria** ICMJE	Inclusion Criteria:<br>• Patients with biopsy verified head and neck squamous cell carcinoma referred for primary curatively intended treatment.<br>• Candidates for concomitant chemoradiotherapy (2Gyx33-34; 6F/W; weekly cisplatin 40mg/m2, max. 70 mg/weekly) according to Danish Head and Neck Cancer Group (DAHANCA) guidelines (T1-4, N1-3, M0)<br>◦ Performance status 0-1<br>◦ At least 18 years of age.<br>Exclusion Criteria:<br>• BMI below 20.5<br>• diabetes<br>• corticosteroid treatment for other diseases

	• Tonsillectomy within the last week before inclusion.<br>• hemoglobin below 6 mmol/l<br>• leucocytes below 2.5 x 10^9 /l<br>• thrombocytes below 50 x 10^9 /l<br>• comorbidities, social, familial or geographical conditions, that could compromise attendance or results
**Gender**	Both
**Ages**	18 Years and older (Adult, Senior)
**Accepts Healthy Volunteers**	No
**Contacts** ICMJE	Contact: Julie Gehl · 004538683868 · karen.julie.gehl@regionh.dk<br>Contact: Camilla K Lonkvist · 004538689571 · camilla.kjaer.loenkvist@regionh.dk
**Listed Location Countries** ICMJE	Denmark
**Removed Location Countries**	

Administrative Information

**NCT Number** ICMJE	NCT02557529
**Other Study ID Numbers** ICMJE	**DAHANCA** 31
**Has Data Monitoring Committee**	No
**Plan to Share Data**	*Not Provided*
**IPD Description**	*Not Provided*
**Responsible Party**	Julie Gehl, Herlev Hospital
**Study Sponsor** ICMJE	Herlev Hospital
**Collaborators** ICMJE	• Aarhus University Hospital<br>• Odense University Hospital<br>• Danish Head and Neck Cancer Group
**Investigators** ICMJE	Principal Investigator: · Julie Gehl · Herlev Hospital
**Information Provided By**	Herlev Hospital
**Verification Date**	May 2016

ICMJE **Data element required by the International Committee of Medical Journal Editors and the World Health Organization ICTRP**
