# Supplementary material for: Progressive resistance training in head and neck cancer patients during concomitant chemoradiotherapy -- design of the DAHANCA 31 randomized trial
Source: BMC Cancer. 2017 Jun 3;17:400. doi: 10.1186/s12885-017-3388-0 (PMC5457597; doi:10.1186/s12885-017-3388-0)
Supplement: Supplementary file 4 — Antiemetic regimens. Antiemetics are given according to institutional guidelines. At Herlev site the regimen changed May 23rd 2016 due to standardization in the Capital Region. Day 1 is the day cisplatin is given. In addition, Domperidon 20–30 mg PRN is administered P.O. a maximum of thrice daily. Abbreviations: p.o., per os. PRN, pro re nata (when necessary). (PDF 14 kb) [file 12885_2017_3388_MOESM4_ESM.pdf]

Additional Table 1. Antiemetic regimen.

	Herlev site prior to May 23rd 2016		Herlev site after May 23rd 2016		Aarhus site	
	Medicine	Dose	Medicine	Dose	Medicine	Dose
Day 1			Palonosetron	0.5 mg P.O.	Palonosetron	0.5 mg P.O.
			Aprepitant	125 mg P.O.	Aprepitant	125 mg P.O.
	Prednisolone	100 mg P.O.	Prednisolone	50 mg P.O.	Prednisolone	50 mg P.O.
	Ondansetron	16-24 mg P.O.				
Day 2			Aprepitant	80 mg P.O.	Aprepitant	80 mg P.O.
	Prednisolone	2 x 50 mg P.O.	Prednisolone	50 mg P.O.	Prednisolone	50 mg P.O.
Day 3					Aprepitant	80 mg P.O.
	Prednisolone	2 x 25 mg P.O.	Prednisolone	50 mg P.O.		
Day 4	Prednisolone	25 mg P.O.	Prednisolone	25 mg P.O.		

Additional Table 1. Antiemetics are given according to institutional guidelines. At Herlev site the regimen changed May 23rd 2016 due to standardization in the Capital Region. Day 1 is the day cisplatin is given. In addition, Domperidon 20-30 mg PRN is administered P.O. a maximum of thrice daily. Abbreviations: p.o., per os. PRN, pro re nata (when necessary).
