# Supplementary material for: Progressive resistance training in head and neck cancer patients during concomitant chemoradiotherapy -- design of the DAHANCA 31 randomized trial
Source: BMC Cancer. 2017 Jun 3;17:400. doi: 10.1186/s12885-017-3388-0 (PMC5457597; doi:10.1186/s12885-017-3388-0)
Supplement: Supplementary file 5 — Illustrations of the exercises. Illustrations of the exercises. (PDF 24 kb) [file 12885_2017_3388_MOESM5_ESM.pdf]

Addtional Figure 1. Illustrations of the exercises

1.	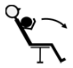	Abdominal crunches (Herlev site)
	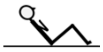	Sit ups (Aarhus site)
2.	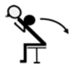	Back extensions
3.	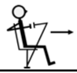	Chest Press
4.	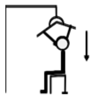	Lateral pull down (Aarhus site)
	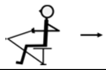	Low row (Herlev site)
5.	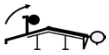	Hamstring curls
6.	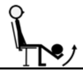	Knee extensions
7.	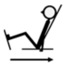	Leg press
